# Effect of Pressure-Assisted Heat Treatment on the Crystalline Phase of Titanium Dioxide, Niobium Pentoxide, and Ruthenium-Modified Oxides

**DOI:** 10.1021/acsomega.5c08955

**Published:** 2026-01-23

**Authors:** Pietra B. Pires, Maria E. K. Fuziki, Giane G. Lenzi, Simone do Rocio F. Sabino, Andressa Novatski, Sergio M. Tebcherani, Daniele Toniolo Dias

**Affiliations:** † Departamento Acadêmico de Engenharia Química, Universidade Tecnológica Federal do Paraná, Campus Ponta Grossa, Ponta Grossa, Paraná 84017-220, Brazil; ‡ Centro de Caracterização Multiusuário em Pesquisa e Desenvolvimento de Materiais, Universidade Tecnológica Federal do Paraná, Campus Ponta Grossa, Ponta Grossa, Paraná 84017-220, Brazil; § Departamento de Física, Universidade Estadual de Ponta Grossa, Ponta Grossa, Paraná 84030-900, Brazil; ∥ Programa de Pós-Graduação em Engenharia de Produção, Universidade Tecnológica Federal do Paraná, Campus Ponta Grossa, Ponta Grossa, Paraná 84017-220, Brazil; ⊥ Departamento Acadêmico de Física, Universidade Tecnológica Federal do Paraná, Campus Ponta Grossa, Ponta Grossa, Paraná 84017-220, Brazil

## Abstract

Titanium dioxide, niobium pentoxide, and ruthenium-modified tungsten were synthesized using sol–gel methodology. The effect of pressure-assisted heat treatment (PAHT) on their structural, morphological, and optical properties was investigated. The X-ray diffraction, scanning electron microscopy, energy-dispersive X-ray, UV–vis diffuse reflectance spectroscopy, and Raman spectroscopy analyses highlighted significant changes in structural, morphological, and optical properties, especially in the phase, band gap energy, and visible light absorption after PAHT. The results indicated that the 100Ti:1Ru oxide, obtained by the sol–gel method, calcined at 800 °C, and subjected to PAHT, presented promising characteristics for chemical reactions activated by solar radiation, with a notable reduction in band gap and visible absorption. Furthermore, PAHT promoted more stable crystalline phases at temperatures much lower than those indicated in the literature related to synthesizing titanium dioxide and niobium pentoxide. These discoveries contribute significantly to the advancement of knowledge, providing valuable guidance for developing and optimizing materials with specific properties for technological applications.

## Graphical abstract

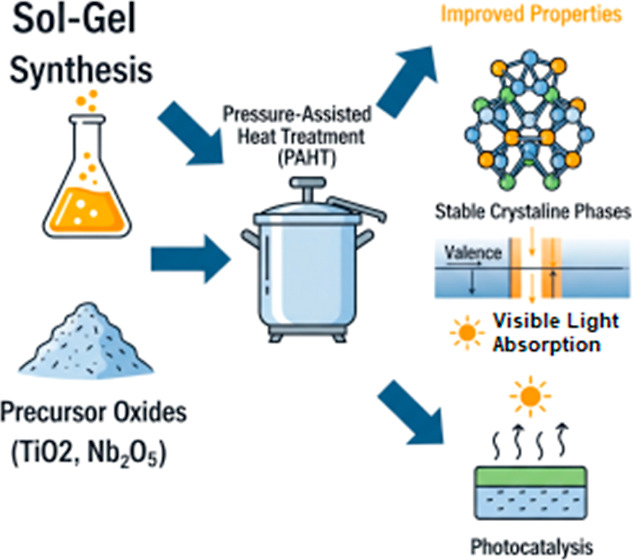

## Introduction

The improvement of semiconductor oxides that are capable of being applied in catalysis, dye-sensitized solar cells, and photocatalysts in water and air systems requires constant advanced research.^1^ In this scenario, the development of modified composites can enhance or even replace compounds widely used for various purposes, such as titanium dioxide (TiO_2_), for the degradation of organic compounds into substances that are less harmful to the environment.^2^ TiO_2_ is known for its remarkable characteristics that make it stand out: nontoxicity; high photochemical activity; low cost; stability in aqueous systems; and chemical stability over a wide pH range.^3^ Furthermore, the development of new photocatalysts with properties similar to or superior to those of titanium dioxide is of paramount importance. In this context, niobium pentoxide (Nb_2_O_5_) has similar properties and is found in large quantities in Brazil, where it holds the largest reserves of this mineral.^4,5^ Dias et al. found promising results in the synthesis of these oxides as stable crystalline structures, visible photoexcitation, and at calcination temperatures lower than those reported in the literature.^6^ As additional features, the addition of doping elements, such as ruthenium (Ru), for example, in oxide materials has the potential to create unique and improved features, as this doping overcomes the difficulty of stabilizing components in the development of new materials.^6,7^

The application of a physical method called pressure-assisted heat treatment (PAHT) to semiconductor oxides can produce modified materials with diverse nanostructures and morphologies. PAHT was developed as a new physical method for thin-film deposition and has become a promising alternative treatment for producing new complex materials, primarily due to the use of low temperatures and high gas pressure (e.g., air).^8–15^ In the deposition field, Cava et al. observed a notable reduction in free energy, which occurred due to the equilibrium transformation from the metastable amorphous state to the crystalline state in the formation mechanism of M_*x*_O_*y*_–SiO_2_ films (using SnO_2_, Co_3_O_4_, TiO_2,_ and Al_2_O_3_ oxide powders).^11^

The development of modified composites is an important point in the synthesis of nanomaterials, which can enhance or even replace compounds widely used for various purposes such as, for example, the use of titanium dioxide (TiO_2_) as a semiconductor for the degradation of organic matter. TiO_2_ is the most used catalyst in oxidative processes mainly due to the following characteristics: commercial availability, photostability, chemical stability in a wide pH range, activation by ultraviolet (UV) sunlight, immobilization on solids, and not presenting toxicity.^16^ This compound has been used in treating industrial and domestic effluents, leachate and gaseous emissions, and sanitary disinfection of water and sewage, with the advantage of not generating toxic byproducts as in other treatments. Other studies demonstrate the bactericidal capacity of heterogeneous photocatalysis with TiO_2_, performing the inactivation of microorganisms.^17^ Furthermore, PAHT on thin films (CCTO, ZnO) deposited by other methods promoted disordered clusters, increasing the photoluminescence (PL) of such materials and a midrange disturbance of the cluster structure after PAHT, causing a redshift in the PL emission of the thin film and consequently a decrease in the band gap.^12,13^ Drabeski et al. demonstrated that PAHT varies the amount of oxygen adsorbed in SNO_2_ films deposited by the spin coating technique, changing the stoichiometry to SnO_*x*_ (with *X* less than 2), and that after PAHT, the absorption band related to SnO_2_ showed a blueshift and consequent increase in the band gap energy.^9^ Studies show that oxide powders also had their PL modified by PAHT, although to a lesser extent when compared to thin films. The rich oxygen atmosphere favors the formation of intermediate levels within the band gap, reducing the energy of the PL emission, which was observed in the redshift in the PL emission after PAHT of inorganic oxide powders (α-Bi_2_O_3_).^14^ Recently, Saito et al. confirmed that PAHT induced small changes in the crystal structure of the Fe_2_O_3_, Cr_2_O_3_, and MoO_3_ samples. Despite this, the band gap values remained similar in these studied oxides.^10^ Da Trindade et al. prepared SnO_2_ samples by the coprecipitation method and demonstrated that PAHT improved the performance of the samples as photocatalysts in dye degradation and negatively interfered with the performance of the samples without prior heat treatment.^15^ Therefore, PAHT can cause structural modifications as well as the creation of various types of defects in crystalline thin films and oxide powders. For example, ordered clusters can be disordered by pressure, leading to significant changes in the materials’ physical and chemical properties. Furthermore, the effect of air pressure on crystalline structures can develop new complex materials with tunable physical and chemical properties for a wide variety of technological applications.

The purpose of this article was to synthesize TiO_2_, Nb_2_O_5_, and ruthenium-modified oxides using the sol–gel method. Here, for the first time, the effect of PAHT on their morphology, structure, and thermo-optical properties will be compared. The X-ray diffraction (XRD), scanning electron microscopy (SEM), energy-dispersive X-ray (EDX) analysis, energy-dispersive spectroscopy (EDS), Raman spectroscopy, and UV–vis diffuse reflectance spectroscopy (DRS) measurements were applied to consider the potential for generating significant discoveries in the development of modified oxides and the strengthening of possible innovative applications.

## Results and Discussion

In a previous article, X-ray diffractometry was used to identify the phases and crystal structures present in the oxides without PAHT and to evaluate possible crystallographic changes caused by changes in the levels of factors involved in oxide synthesis, such as increased calcination temperature and doping.^6^ In this work, XRD was applied to analyze the influence of PAHT on the crystal structure of the solid solution oxides and identify possible phase changes. Therefore, Figure 1 presents the X-ray diffractograms of the oxides as a function of their dopant percentage (0% and 1% Ru) and calcination temperatures (400 and 800 °C) after PAHT.

**Figure 1:**
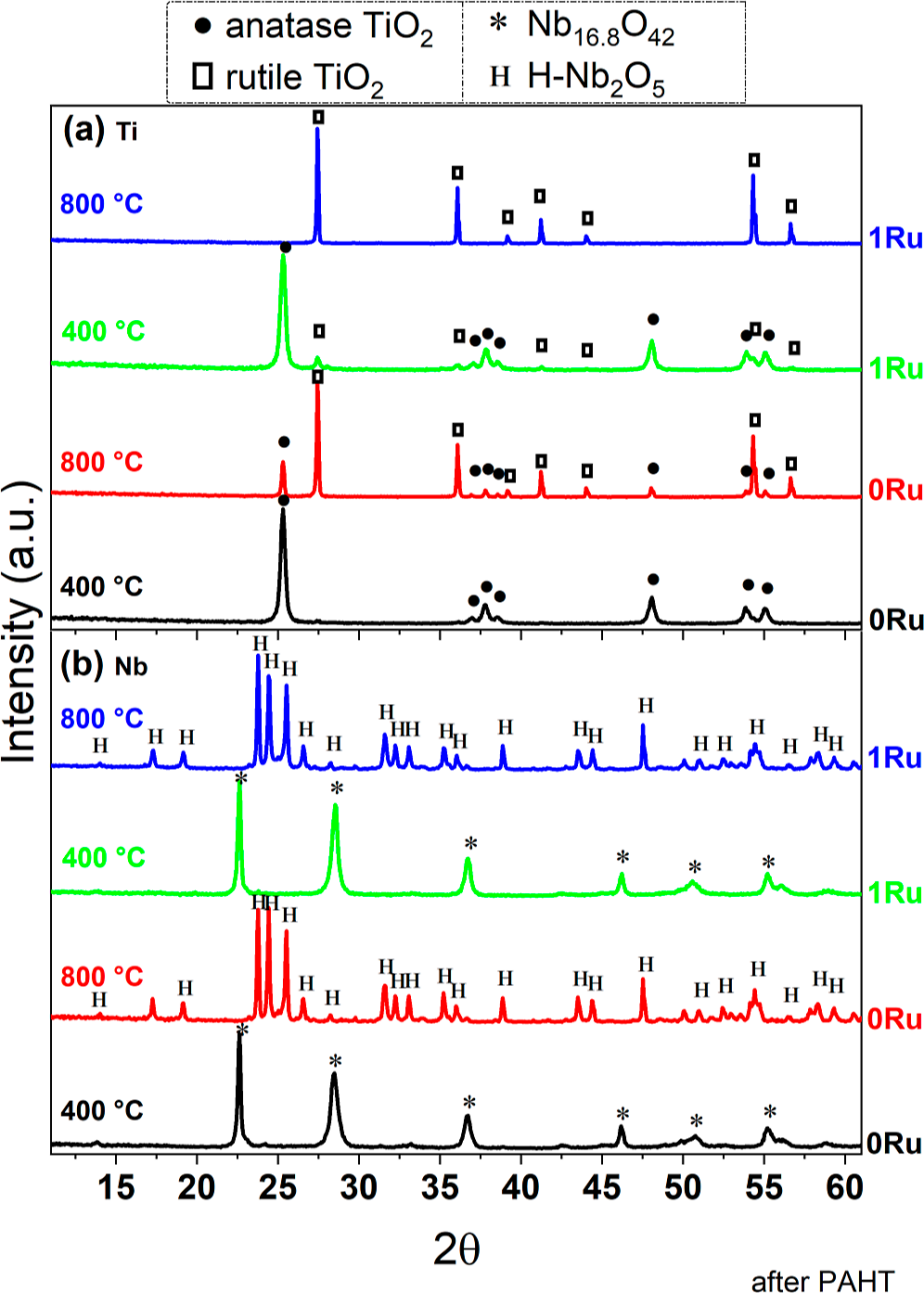
XRD patterns of the 0% Ru and 1% Ru samples calcined at 400 and 800 °C for the oxides after PAHT with (a) TiO_2_ and (b) Nb_2_O_5_. XRD analysis revealed the formation of stable phases even at a lower temperature level and a change in the crystallographic phase.

Table 1 is a compilation of results from X-ray analysis and the crystal structure refinement using the Rietveld method (Figure S1 and Table S1) of the oxides without PAHT, previously cataloged^6^ and, for comparison purposes, after PAHT. Analysis of Table 1 reveals the influence of calcination temperature on the formation of crystallographic phases as well as possible alterations resulting from the application of PAHT.

**Table 1:** Parameters Obtained after Rietveld Refinement for Oxides Calcined at 400 and 800 °C without PAHT (wP) and after PAHT (aP)

sample code	*a* (Å)	*b* (Å)	*c* (Å)	*V* (Å^3^)	phase	wt % (%)
Ti:0Ru 400 wP	3.791312	3.791312	9.527563	136.95	anatase	100.0
Ti:0Ru 400 aP	3.780617	3.780617	9.507156	135.886	anatase	100.0
Ti:0Ru 800 wP	3.783825	3.783825	9.513948	136.214	anatase	22.6
	4.593354	4.593354	2.959232	62.437	rutile	77.4
Ti:0Ru 800 aP	3.784133	3.784133	9.51092	136.193	anatase	20.8
	4.592679	4.592679	2.958581	62.404	rutile	79.2
Ti:1Ru 400 wP	3.786985	3.786985	9.51254	136.422	anatase	87.4
	4.592739	4.592739	2.960654	62.452	rutile	12.6
Ti:1Ru 400 aP	3.784834	3.784834	9.505736	136.184	anatase	90.0
	4.589343	4.589343	2.962986	62.407	rutile	10.0
Ti:1Ru 800 wP	4.593258	4.593258	2.960092	62.452	rutile	100.0
Ti:1Ru 800 aP	4.592173	4.592173	2.959627	62.413	rutile	100.0
Nb:0Ru 400 wP	6.211788	29.08646	3.924934	709.1528	TT-Nb_2_O_5_	100.0
Nb:0Ru 400 aP	6.217876	29.10761	3.929489	711.1885	TT-Nb_2_O_5_	100.0
Nb:0Ru 800 wP	21.19783	3.826504	19.39201	1364.805	H-Nb_2_O_5_	100.0
Nb:0Ru 800 aP	21.17334	3.823112	19.36802	1360.111	H-Nb_2_O_5_	100.0
Nb:1Ru 400 wP	6.230186	29.02805	3.927274	710.248	TT-Nb_2_O_5_	100.0
Nb:1Ru 400 aP	6.232354	29.05142	3.92972	711.51	TT-Nb_2_O_5_	100.0
Nb:1Ru 800 wP	6.224107	29.04521	3.928145	710.132	TT-Nb_2_O_5_	100.0
Nb:1Ru 800 aP	21.16885	3.823457	19.36116	1359.526	H-Nb_2_O_5_	100.0

It was not possible to identify a phase related to Ru in the XRD diffractograms, which may indicate its dispersion in the structures of the TiO_2_ and Nb_2_O_5_ phases. For TiO_2_, the presence of Ru caused interesting changes in the lattice parameters and phase transition of the material, notably favoring the formation of the rutile phase. Considering the Rietveld Refinement performed on the XRD diffractograms of TiO_2_ heat-treated under different conditions (Table 1), it was possible to observe that the introduction of ruthenium, at 400 °C, favored the formation of a small rutile portion even at low temperatures. In the absence of PAHT, the dopant incorporation led to a decrease in the lattice parameters of the anatase phase calcined at 400 °C (Ti:1Ru 400 wP). On the other hand, with the implementation of PAHT, the introduction of ruthenium led to a slight increase in anatase lattice parameters (Ti:1Ru 400 aP). At a temperature of 800 °C, the introduction of ruthenium led to complete conversion to rutile, as well as an increase in cell volume and a distortion in the lattice of the rutile phase, with a slight reduction in parameters a and b and an elongation in parameter c, regardless of PAHT. For Nb_2_O_5_, the incorporation of Ru into the oxide did not affect the phase observed at 400 °C, but it did promote alterations in the lattice parameters and cell volume (Nb:1Ru 400 wP). At 800 °C, the addition of ruthenium prevented the formation of the monoclinic phase in the samples not treated with PAHT (Nb:1Ru 800 wP), requiring the application of PAHT to promote this phase again (Nb:1Ru 800 aP). Still, it only caused minor changes in the lattice parameters when PAHT was performed in the base oxide (Nb:0Ru 800 aP).

Regarding PAHT treatment, its application to TiO_2_ led to a reduction in cell volume in all phases observed in the samples, although a few parameters showed a slight increase. Even though the treatment did not affect the TiO_2_ phases obtained, it changed the phase proportion, favoring the formation of rutile at 800 °C without the incorporation of ruthenium into the oxide and favoring the formation of anatase for the catalyst treated at 400 °C with the addition of Ru. In the case of niobium oxide, PAHT promoted an increase in lattice parameters for materials calcined at 400 °C. However, for materials calcined at 800 °C, the treatment led to a reduction in the lattice parameters of the oxide prepared without the addition of Ru. In turn, it resulted in a phase change to monoclinic in the case of the oxide prepared with ruthenium addition.

It is worth noting that the sol–gel synthesis methodology used in this work may have induced pores and increased the surface area, requiring a lower calcination temperature, compared to the literature, to form more stable structures in the material’s morphology.^4,18,19^ Therefore, the stable phases of both precursor materials (Ti and Nb) at higher temperatures, combined with the insertion of ruthenium and application of PAHT, cause crystallographic changes and stability, possibly altering the optical characteristics of the material, even at lower calcination temperatures.

Figure 2 shows the morphological differences of TiO_2_ particles (Figure 2a,b), Nb_2_O_5_ (Figure 2e,f), and their respective modified samples (Figure 2c,d; Figure 2g,h), calcined at 400 and 800 °C, and after PAHT, characterized by SEM. The images formed by the oxides after PAHT showed smoother and more regular surfaces with larger and brighter grains than the images and EDS spectrum of the oxides without PAHT, published elsewhere.^6^ In addition, EDX mapping analyses for oxides without PAHT can be evaluated in Figure S2. Lines 2–4 show EDX elemental maps of O, Ti or Nb, and Ru to identify the distribution of the different elements within representative particles. Furthermore, the O, Ti, Nb, and Ru atoms are homogeneously dispersed on the surface of all oxides, as shown in the SEM–EDX mapping images of Figure S2 (without PAHT) and Figure S3 (after PAHT). Unlike TiO_2_ oxides, the element ruthenium cannot be detected in Nb_2_O_5_ oxides at either calcination temperature, as can be seen in line 4 of Figures S2 and S3. It is possible that the element Ru, in addition to its low concentration, is highly dispersed in the Nb_2_O_5_ matrix and, therefore, may be below the detection limit of EDX. The element ruthenium is difficult to detect in a niobium pentoxide matrix by EDX primarily due to the higher background signal and potential peak overlaps associated with the heavier Nb matrix. A sample with a higher mean atomic number, such as Nb_2_O_5_, produces a much higher X-ray continuum (background) compared to a lower atomic number matrix like TiO_2_. This elevated background noise in Nb_2_O_5_ makes distinguishing the smaller peaks from trace amounts of Ru much harder statistically, often falling below the minimum detection limit.^6^ In the TiO_2_ matrix, the lower background allows the Ru peak to be resolved more easily.^6^ EDS analysis showed, for all oxides, that energy peaks of other chemical elements were not generated on the surfaces of the particles of the respective materials analyzed beyond the expected titanium, niobium, and elemental oxygen (as can be seen in Figure S4). Therefore, the chemical composition data by EDS on the surfaces of particles of the samples without modification by ruthenium (Figure S4a,b,e,f) suggest that the sol–gel synthesis produced a base with high purity at the two levels of calcination temperature used in the synthesis. As previously discussed for EDX mapping, when 44Ru anchoring occurs with the lighter element 22Ti, it is possible to generate an energy peak referring to the element ruthenium without overlapping the Ti peak, indicating its presence at both calcination temperatures (Figure S4c,d). On the other hand, both energy peaks were generated superimposed, for the Nb:1Ru sample (see Figure S4g,h), highlighting the percentage of the element 41Nb compared to 44Ru in the surface chemical composition of the particles of this oxide.

**Figure 2:**
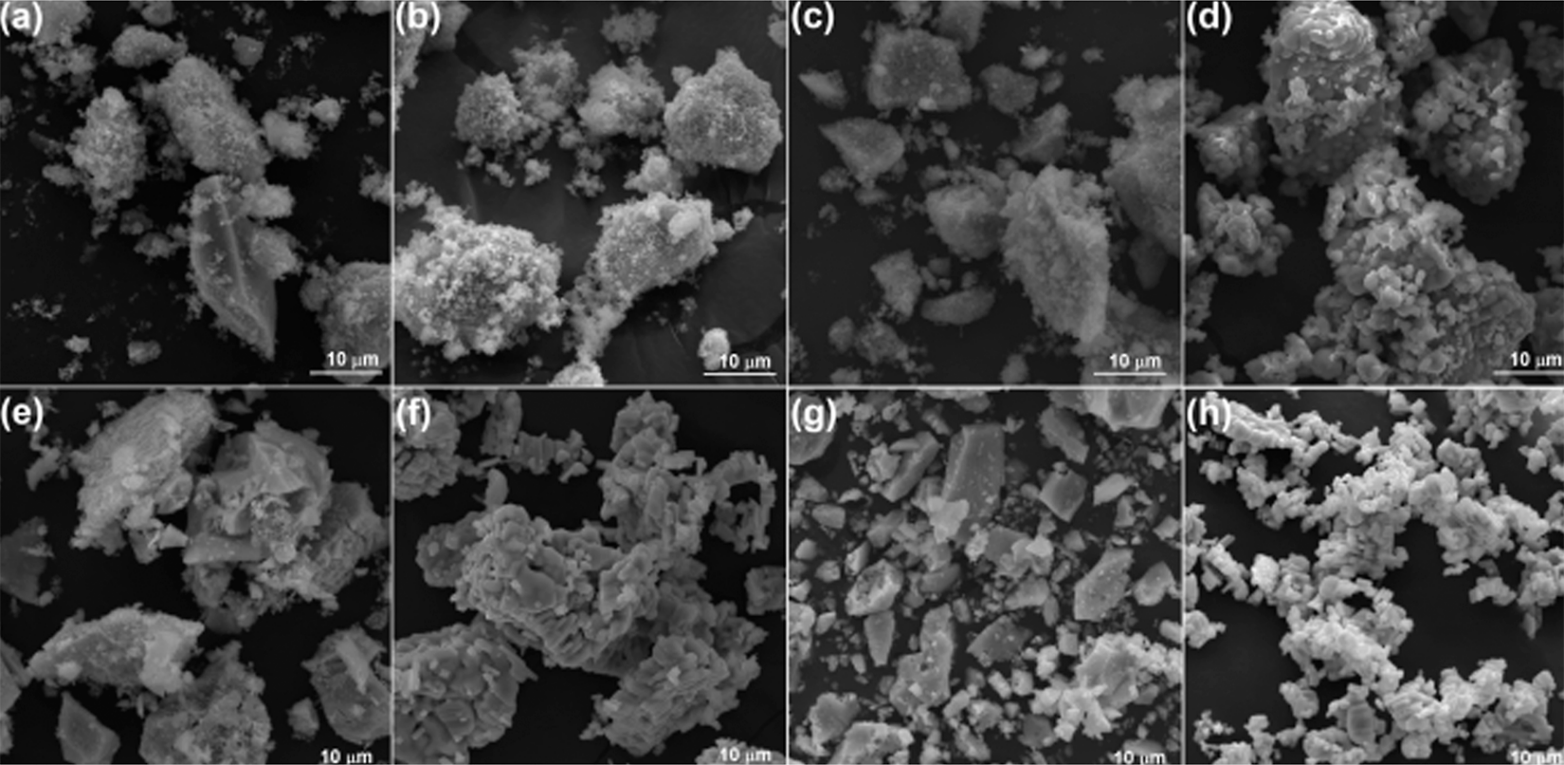
SEM images (5k× magnification) for the base oxides of TiO_2_, Nb_2_O_5_, and ruthenium-modified oxides after PAHT. (a,e) Calcined base samples, i.e., Ti:0Ru, and Nb:0Ru at 400 °C of calcination temperature. (b,f) Base samples at 800 °C of calcination temperature. (c,g) Calcined modified samples, i.e., Ti:1Ru, and Nb:1Ru 400 °C of calcination temperature. (d,h) Modified samples at 800 °C of calcination temperature.

The micrographs of the titanium dioxide-based samples, shown in Figure 2a,b, revealed a uniform distribution of irregular titanium dioxide crystals with many particles of varying diameters. However, when the sample was subjected to a higher level of calcination, the micrograph in Figure 2b showed a less defined appearance of the material with a denser and more granular texture. These characteristics are similar to the surface morphologies of the anatase and rutile phases of synthesized TiO_2_.^20,21^ Modification with ruthenium at the upper calcination stage (Figure 2d) promoted the growth of larger granules compared to the base oxides, presenting a smoother surface and with greater porosity. Bright particles aggregated into the larger structures were also observed.

In the micrographic images of the niobium pentoxide samples calcined at the lower temperature level (Figure 2e,g), a homogeneous distribution of particles and a dense and smooth texture were observed with a smaller presence of agglomerates around the crystals. This morphology resembles the surface shape of orthorhombic niobium oxide (T-Nb_2_O_5_).^22^ Figure 2g reveals that the ruthenium modification formed denser clusters with the presence of some vacancies (Moon-like). However, the most evident change occurred for the samples calcined at the higher temperature level and subjected to PAHT (Figure 2f,h). The micrographs exhibited dispersed clusters of different sizes, resembling the surface morphology of the monoclinic niobium oxide (H-Nb_2_O_5_).^4,23,24^ The influence of PAHT on ruthenium-doped samples was also more evident in this case (Figure 2h), in which the Nb_2_O_5_ crystals were more defined, with a surface formed by particle agglomerates well-delineated and more scattered, very different from the smooth morphology observed in the samples without PAHT (Figure S1h).

In accordance with the XRD analyses (Figure 1 and Table 1), Figure 3 shows the Raman spectra for all oxides without PAHT (wP in Figure 3a,b) and after PAHT (aP in Figure 3c,d). The spectra indicated the high crystallinity of the oxides under study, providing sharp peaks with very low fluctuation in the measurement. This high crystallinity is related to the atomic order of the oxide structures, which can directly influence their physical and chemical properties. Note that the increase in the synthesis temperature of the Ti and Nb oxides, as well as the application of TTAP, resulted in significant changes in the crystalline phase of the materials. These changes were accompanied by changes in the intensities and positions of the Raman peaks.

**Figure 3:**
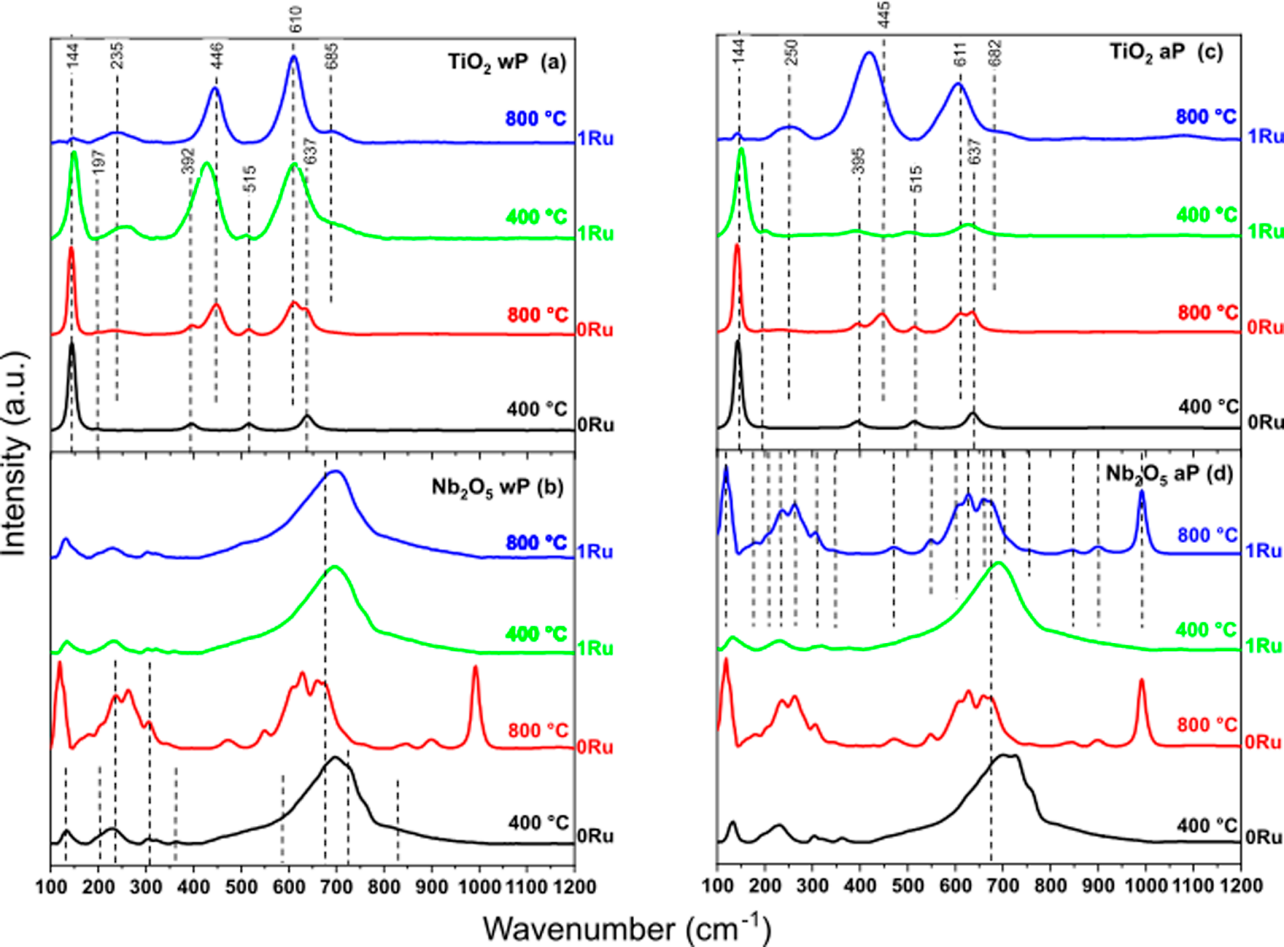
Raman spectra of the TiO_2_, Nb_2_O_5_, and ruthenium-modified oxides powders calcined at temperatures of 400 and 800 °C: (a,b) without PAHT (denoted by wP) and (c,d) after PAHT (denoted by aP).

In Figure 3a, the TiO_2_ base oxide calcined at 400 °C presented the five allowed modes for the anatase phase: 144 cm^–1^ (E_g_), 197 cm^–1^ (E_g_), 392 cm^–1^ (B_1g_), 515 cm^–1^ (A_1g_), and 637 cm^–1^ (E_g_).^25^ Increasing the calcination to 800 °C promoted the anatase–rutile phases. Therefore, in addition to the five modes allowed for the anatase phase 143 cm^–1^ (E_g_), 197 cm^–1^ (E_g_), 396 cm^–1^ (B_1g_), 514 cm^–1^ (A_1g_), and 637 cm^–1^ (E_g_), four other energy changes were also observed that are related to the rutile phase,^26^ some superimposed on those of the anatase phase: 143 cm^–1^ (B_1g_), 235 cm^–1^ (scattering), 446 cm^–1^ (E_g_), and 611 cm^–1^ (A_1g_). When doping with ruthenium occurs, the sample calcined at 400 °C also presented the anatase phases at 148 cm^–1^ (E_g_), 233 cm^–1^ (E_g_), 392 cm^–1^ (B_1g_), 507 cm^–1^ (A_1g_), 514 cm^–1^ (B_1g_), and 656 cm^–1^ (E_g_) and rutile at 148 cm^–1^ (B_1g_), 258 cm^–1^ (scattering), 423 cm^–1^ (E_g_), and 615 cm^–1^ (A_1g_), with a certain shift of the peaks. The E_g_ modes for anatase TiO_2_ underwent a blueshift (to higher wavenumbers) with ruthenium anchoring. These vibration modes are particularly sensitive to the presence of oxygen vacancies, and a blueshift indicates a decrease in these. Therefore, the base sample Ti:0Ru 400 has a high oxygen deficiency and appears lighter in color than Ti:1Ru 400, as seen in Table 2.

**Table 2:**
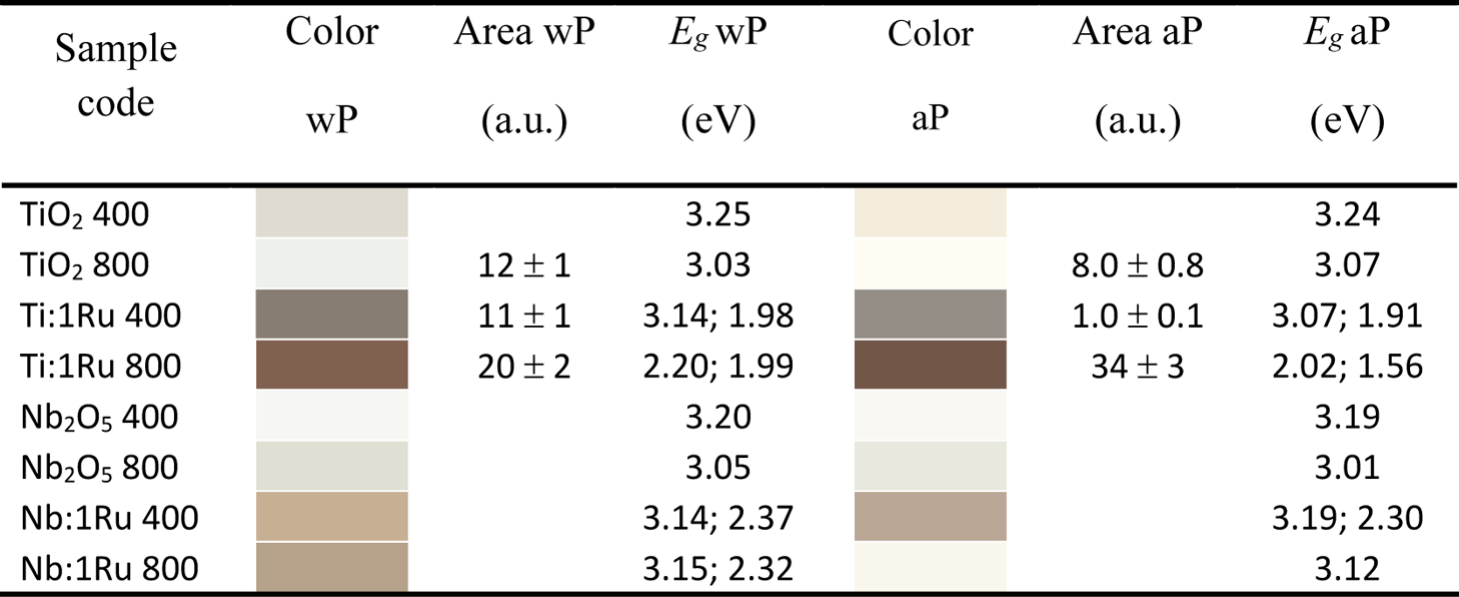
Oxides Developed by Sol–Gel Methodology Calcined at 400 and 800 °C^c^

a *E*_g1_: 1st gap.

b *E*_g2_: 2nd gap.

c Color, area under the curve at ≈ 446 cm^–1^ (*E*_g_ mode rutile-TiO_2_), and *E*_g1_ [Table-fn t2fn1] and *E*_g2_ [Table-fn t2fn2] estimated by UV–vis DRS without PAHT (wP) and after PAHT (aP).

On the other hand, the Ru-doped oxide calcined at 800 °C presented only the rutile phase. In the latter case, the characteristic bands of rutile TiO_2_ are observed at 235, 438, and 610 cm^–1^, which can be attributed to the multiple photon scattering process and the Raman-active modes E_g_ (planar O–O vibration) and A_1g_ (Ti–O stretching), respectively. In addition, the small bands at 148 and 870 cm^–1^ are assigned to E_g_ and B_2g_. Note that, for the ruthenium-doped TiO_2_ oxides, the planar O–O vibration and Ti–O stretching bands are broadened compared to the base since the characteristic ruthenium bands are possibly superimposed in these two regions. When performing a peak separation (see Figure 4), three superimposed bands referring to a rutile phase of RuO_2_ are observed: 444 cm^–1^ (E_g_), 584 cm^–1^ (Ru^3+^–O), and 706 cm^–1^ (Ru^4+^–O) for the oxide calcined at 400 °C (Figure 4a) and 446, 579, and 685 cm^–1^ for the sample doped at 800 °C (Figure 4b). Table 2 also shows the area under the curve at ≈ 446 cm^–1^ for the E_g_ mode rutile-TiO_2_. It is possible to observe an increase in the area of this peak for Ti:1Ru 800, indicating an increase in the level of oxygen.

**Figure 4:**
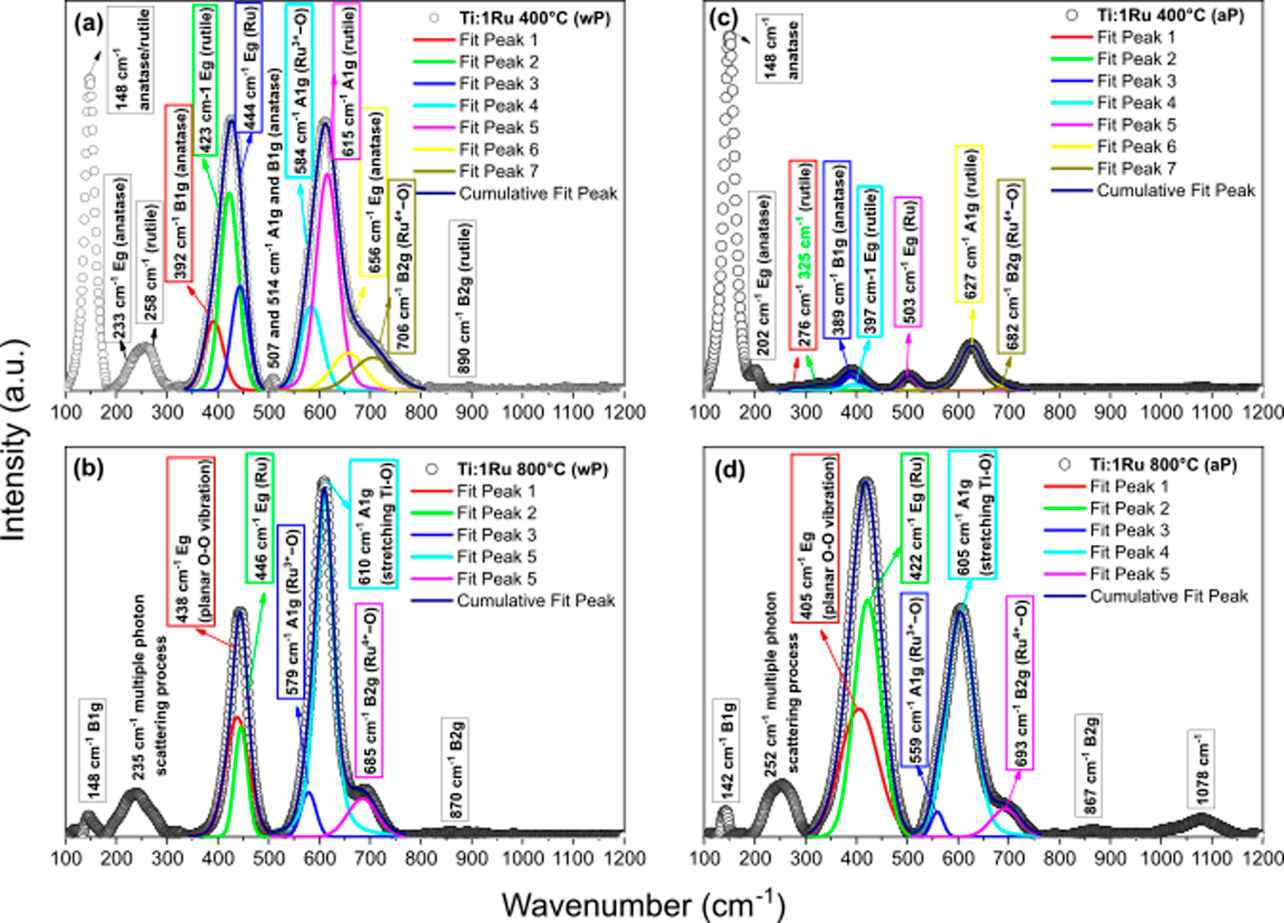
Band separation indicating the possible rutile RuO_2_ phase for Ti:1Ru oxides calcined at (a,c) 400 and (b,d) 800 °C. (a,b) Without PATH (wP), and (c,d) after PAHT (aP).

The Raman analysis for niobium pentoxide, presented previously in Figure 3b, also indicated that the increase in calcination temperature promoted the change of the crystalline phase in the base oxides: from the orthorhombic phase (T-Nb_2_O_5_) to the monoclinic phase (H-Nb_2_O_5_).^4^ The bands in the wavenumber region of 400–800 cm^–1^ are attributed to the symmetric and antisymmetric stretching mode of the Nb–O–Nb bond. The Raman band at 235 cm^–1^ becomes the bending mode of Nb–O–Nb. Graça and coauthors (2013) analyzed Nb_2_O_5_ samples synthesized by the sol–gel method at various calcination temperatures and the Raman spectra of the samples treated at 600 and 800 °C were very similar to each other, with a main vibration mode at ∼690 cm^–1^ related to NbO_6_ octahedral vibrations with low distortion and other modes at ∼220 and ∼310 cm^–1^, corresponding to the T-Nb_2_O_5_ phase (orthorhombic structure). In these authors’ synthesis, only samples heat-treated at higher temperatures than those employed here (900, 1000, and 1200 °C) revealed well-defined Raman spectra characteristic of the monoclinic structure of H-Nb_2_O_5_, presenting an active mode centered at ∼990 cm^–1^ related to the stretching modes of the superficial terminal Nb=O bonds in octahedral NbO_6_ with a higher degree of distortion (structural rigidity).^4^ Generally, one of the most stable polymorphs of Nb_2_O_5_, H-Nb_2_O_5_ (monoclinic phase), can be obtained by heat treatments of niobium oxide at temperatures above 950 °C under air conditions.

Similarly, to the TiO_2_ sample, ruthenium-doped Nb_2_O_5_ presented a broader band, in the region of 400–1000 cm^–1^, when compared to its base. After band separation, the rutile phase bands of ruthenium dioxide were cataloged, centered at 708 and 700 cm^–1^ (E_g_), 784 and 787 cm^–1^ (Ru^3+^–O), and 818 and 824 cm^–1^ (Ru^4+^–O) for the oxides calcined at 400 and 800 °C, respectively. Figure 5 shows the centers of these bands estimated after peak separation in the region of 350–1000 cm^–1^. Note how at 800 °C calcination temperature, a redistribution occurred between the Nb–O–Nb, NbO_6_, and Nb–O bonds to anchor the ruthenium (comparing Figure 5a with [Fig fig5]b). This redistribution also occurred after PAHT to the T-Nb_2_O_5_ phase (Figure 5c). However, it was not possible to separate the rutile phase bands of ruthenium dioxide from the H-Nb_2_O_5_ phase formed after PAHT (Figure 5d).

**Figure 5:**
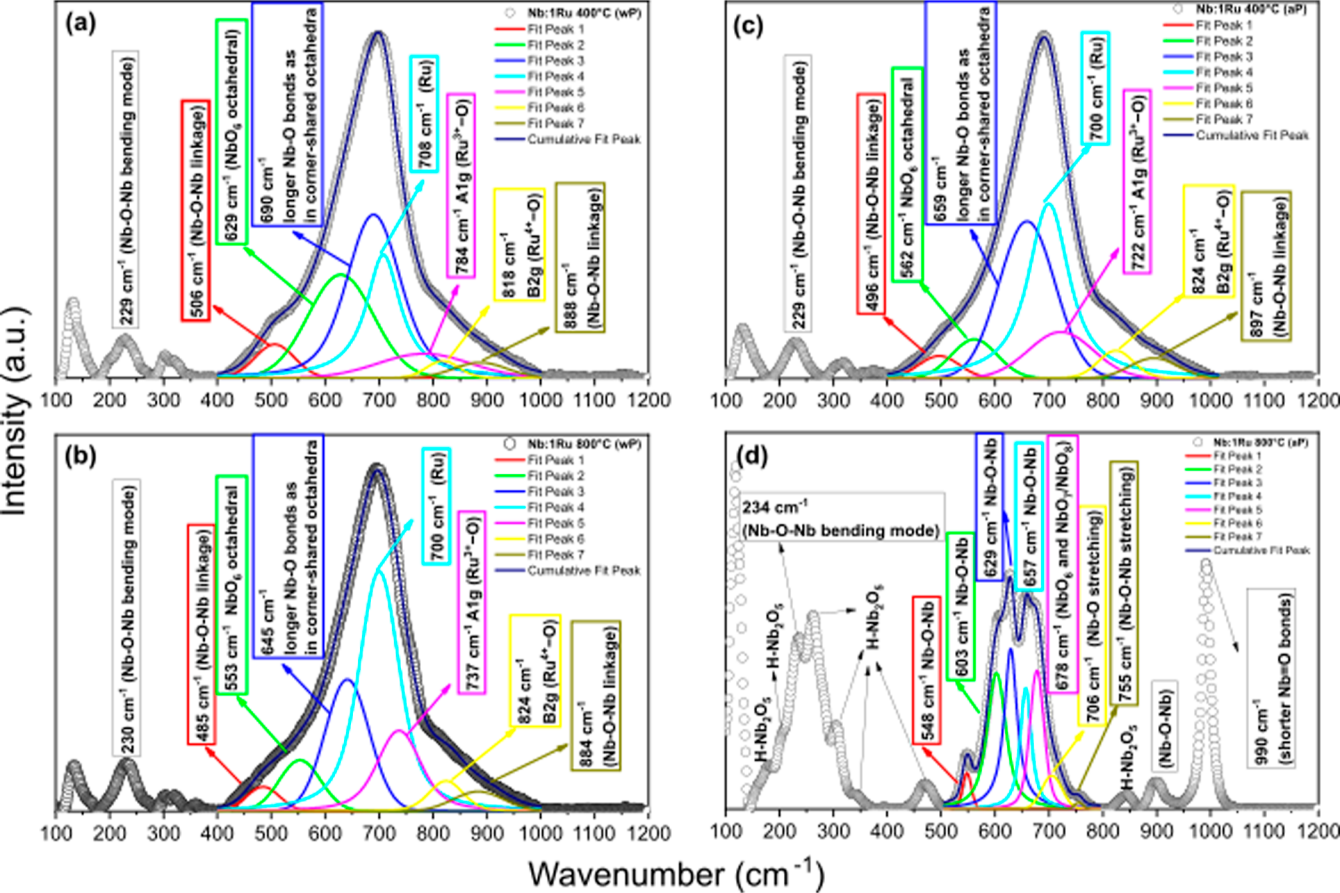
Band separation indicating the possible rutile RuO_2_ phase for Nb:1Ru oxides calcined at (a,c) 400 and (b,d) 800 °C. (a,b) Without PATH (wP), and (c,d) after PAHT (aP).

The presence of additional Raman bands in the 400–500 cm^–1^ region for Nb_2_O_5_ may promote an increase in the oxygen deficiency. This may occur because oxygen vacancies create structural distortions, which are reflected in the vibrational modes detected by Raman spectroscopy. Analyzing the peaks in Figure 5a,b, the band most sensitive to the presence of vacancies appears to be the main vibrational mode at ≈685 cm^–1^, which is attributed to the octahedral coordination of NbO_6_ with low distortion.^27^

Analyzing the Raman spectra in Figure 3 after PAHT, comparing Figure 3a with [Fig fig3]c for the Ti:0Ru oxide at 400 °C, we can see that PAHT had almost no effect on the anatase crystalline phase. Only a slight increase in intensity for high wavenumbers (800–1100 cm^–1^) may indicate a slight structural disorder and the presence of defects such as oxygen deficiency. This is consistent with the refinement results in Table 1, which showed a decrease in lattice parameters, making the material slightly denser after PAHT. On the other hand, a decrease in the intensity of several peaks of the base oxides calcined at 800 °C was observed after PAHT (comparing Figure 3a with [Fig fig3]c,b with [Fig fig3]d), suggesting that less elongated and, therefore, less flexible crystalline structures (such as rutile and H-Nb_2_O_5_) were the most affected by PAHT. In this context, an increase in the ordering of the rutile lattice and consequent increase in crystallinity after PAHT can be perceived for the oxide calcined at 800 °C by the reduction in the intensity of the band around 235 cm^–1^ (Figure 3c), supposedly induced by a second-order scattering process or latent disharmony.^28^ According to Table 1, there was an increase of almost 2 wt % % for the rutile phase after PAHT (Ti:0Ru 800 aP). When comparing the Raman results before and after PAHT, and since, in this case, the samples were not resynthesized, it is possible to rule out how the TiO_2_ powders were oxidized for their formation. Thus, the inversion in the intensity of the bands related to the phonons of the rutile structure 600 (A_1g_) and 418 cm^–1^ (E_g_) is conditioned by the regular occurrence of planar defects in rutile due to oxygen deficiency.^28^ With the ruthenium doping agent, the Ti:1Ru oxide at 400 °C after PAHT undergoes a large reduction in the bands related to the rutile phase (also according to Table 1) and a possible oxygen deficiency (Table 2, the area under the curve at ≈446 cm^–1^). On the other hand, the sample calcined at 800 °C reduces A_1g_ and shows an increase and broadening of the E_g_ band, representing an increase in the O/Ti ratio and consequently in the oxidation of this oxide after PAHT. For the well-developed crystal structure of T-Nb_2_O_5_ at 400 °C (Figure 3d), there was a subtle increase in the bands in the 100–400 cm^–1^ region, attributed to the symmetric and antisymmetric stretching modes of the Nb–O–Nb bond, as well as the bending mode (at 235 cm^–1^). In contrast, the base calcined at 800 °C showed a reduction in peaks except for the first, which remained stable. These oxides showed an increase (Nb:0Ru 400 aP) and a decrease (Nb:0Ru 800 aP) in lattice parameters, as shown in Table 1. In the phase less affected by PAHT formed at 400 °C (T-Nb_2_O_5_), there is a shoulder at 755 cm^–1^, but it is much larger than that in the region centered at 1000 cm^–1^, suggesting longer bond lengths and lower structural rigidity than for the H-Nb_2_O_5_ phase (800 °C). The heat-treated ruthenium-modified samples also showed distinct behaviors depending on the calcination temperature. The lower calcination temperature level showed stability of the Raman peaks after PAHT, with a slight decrease in the intensities of some Nb–O–Nb (stretching mode) Raman peaks. In return, the upper level clearly showed a phase change (from T-Nb_2_O_5_ to H-Nb_2_O_5_) with the formation of intense peaks in the region 150–350 cm^–1^ and less intense ones from 400 to 700 cm^–1^, as well as the emergence of the sharp peak at approximately 1000 cm^–1^, characteristic of short Nb=O bonds. Therefore, the insertion of ruthenium possibly caused less mobility to the crystal structures of the oxide calcined at 800 °C, which enabled the phase change. This insight can be supported by the analysis of Figure 5b, which shows a significant increase in the Ru^3+^–O band (700 cm^–1^) at the expense of long-chain Nb–O and NbO_6_ (533 and 645 cm^–1^). Table 1 also shows a decrease in the unit cell volume (compare the oxides Nb:1Ru 400 wP and Nb:1Ru 800 wP).

Table 2 shows the color hue of the oxides based on the variation in UV–vis DRS measurements. The oxides ranged from very light, subtle grayish (*X* = 91.9232, *Y* = 97.0577, *Z* = 97.1320 for Ti:0Ru 800 aP) to very dark brown (*X* = 11.4390, *Y* = 10.7671, *Z* = 7.5949 for Ti:1Ru 800 aP). The color of the oxide depends on the amount of oxygen present, that is, its oxidation state. TiO_2_ compounds with a predominance of the rutile phase tend to be darker, as they present greater oxidation due to inhibition of the level of oxygen vacancies for their formation. Therefore, PAHT on Ti:1Ru 800 oxide may have inhibited the oxygen vacancy level, promoting the transformation to a more stable oxidation state. In this case, note the inversion in the intensity of the 600 (A_1g_) and 418 cm^–1^ (E_g_) bands, comparing the fourth spectrum in Figure 3a with that in Figure 3c. Except for this oxide, all other oxides appeared lighter after PAHT. Possibly, for these oxides, PAHT increased the oxygen vacancy level, inhibiting the complete transformation of the anatase phase into rutile (for TiO_2_, agreeing with the wt % analysis in Table 1), decreasing oxidation, or promoting a crystalline phase change (for Nb_2_O_5_). In this context, the oxide Nb:1Ru 800 stands out, having changed from the T-Nb_2_O_5_ crystalline phase to H-Nb_2_O_5_, the more thermodynamically stable one. Compare the fourth spectrum in Figure 3b with that in Figure 3d. Note also the drastic change in color of this oxide, from light brown (*X* = 36.8322, *Y* = 37.6025, *Z* = 29.3142) to very light and subtle grayish (*X* = 86.7872, *Y* = 91.7463, *Z* = 91.5778).

The direct band gap values of the oxides without PAHT and after PAHT are shown in Table 2 and Figure 6. The band gap energy (*E*_g_) was estimated by UV–vis DRS spectra and plotted (α*h*ν)­2 against photon energy (*h*ν) using the Tauc method, according to Equation

1 $${\left(\alpha \left(h\nu \right)\right)}^{2}=A\left(h\nu -E_{g}\right)$$

where α is the absorption coefficient, *h* is the Planck constant, and *A* is a constant independent of the frequency ν.^29^ Figure 6 shows the trend of the estimated value for the band gap energy, for the samples calcined at 400 and 800 °C without PAHT (symbols: open square first gap; and closed square second gap) and after TTAP (symbols: open circle first gap; and closed circle second gap). Therefore, the smooth and continuous curved lines in Figure 6 touch the first and last points and are “pulled” by intermediate points for the first gap, and in an isolated way for the second gap (observed only in the Ru-doped samples).

**Figure 6:**
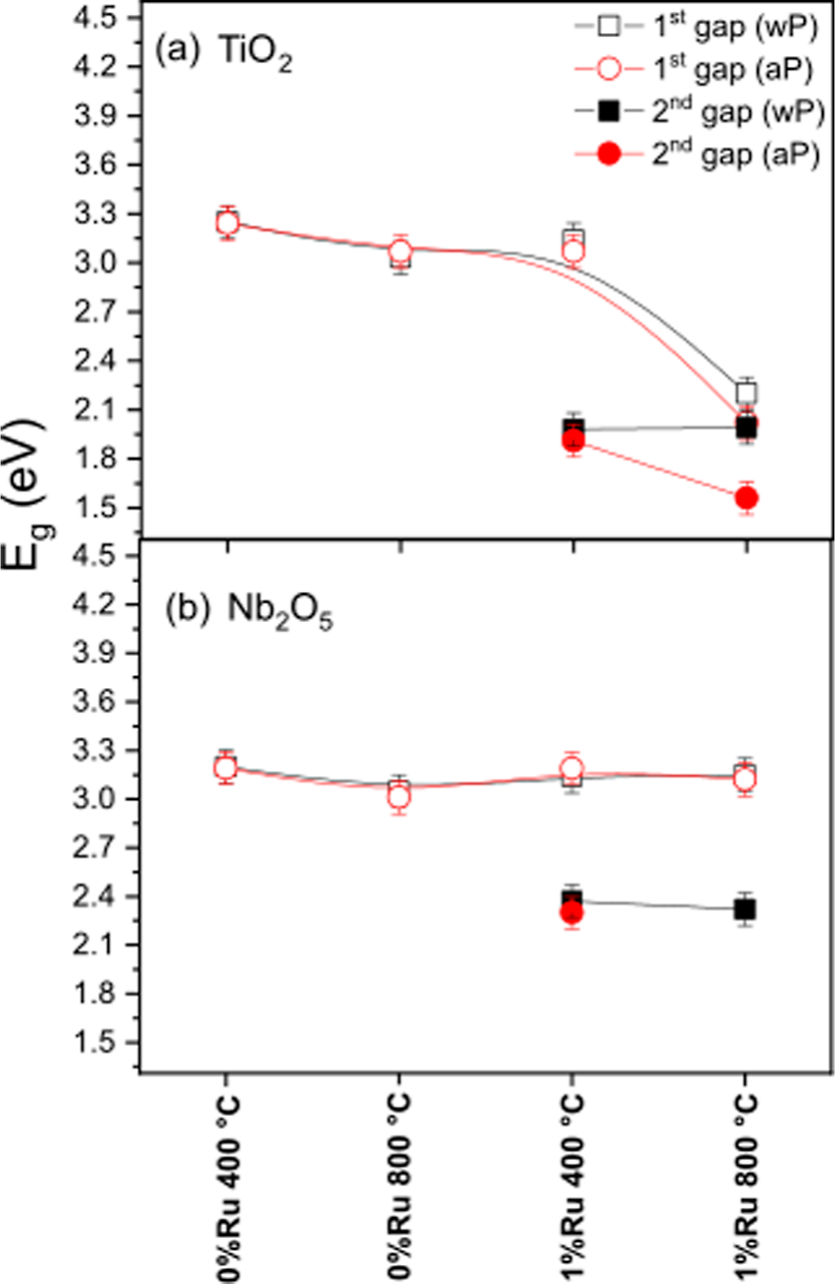
Gap trend and threshold absorption trend for oxides: (a) TiO_2_; and (b) Nb_2_O_5_. The 2nd (second) gap with a value smaller than the 1st (first) gap would be located between the valence band and the conduction band.

In general, the oxides presented a band gap variation from 3.25 to 1.56 eV for TiO_2_ (Figure 6a) and from 3.20 to 2.30 eV for Nb_2_O_5_ (Figure 6b), in agreement with literature data.^5,30–34^ It is known that increasing the temperature promotes high coordination symmetry and stable crystalline phases as a function of the increase in the calcination temperature. TiO_2_ may present a change from the anatase to rutile phase and a consequent decrease in the band gap.^6,35^ Likewise, Nb_2_O_5_ tends to present greater crystallinity at higher temperatures.^4,36^ The points with closed symbols in Figure 6, corresponding to a second band gap for the ruthenium-doped samples, were smaller than the first band gap, as it lies between the valence band (VB) and the conduction band (CB) of the material and is likely related to the transitions of this metal. These values are lower than those found by Mihai et al. (2019) for Ru_*x*_Ti_1–*x*_O_2_ rutile nanostructures (*x* = 0; 0.01; 0.07; 0.16).^37^ After PAHT (closed symbols), the second band gap was the most affected for the samples calcined at 800 °C. This was particularly true for the Nb:1Ru sample, which did not exhibit a second band gap (Figure 6b). Furthermore, PAHT caused a redshift and consequent decrease of approximately 12% for the first band gap and 10% for the second band gap in the Ti:1Ru 800 sample (Figure 6a). The rich oxygen atmosphere favors the formation of intermediate levels inside the band gap, providing a redshift in the absorption band and reducing the band gap after PAHT. A redshift means the presence of band absorption at higher wavelengths (lower energies), indicating the transition of an electron from VB to the CB band is, in part, facilitated. This behavior indicates that air pressure can lead to medium-range disorder in different crystalline structures. This fact can change the defect states inside the band gap and can favor the formation of new interstitial oxygen in the crystalline structures. These results agree with the Raman analyses for this oxide, which showed a redshift from 423 cm^–1^ to 397 cm^–1^ (Figure 4) and oxygen vacancy inhibition (Table 2). Furthermore, the lowest band gap energies were observed for the Ti oxides that showed the highest wt % for the rutile phase in Table 1.

In the context of oxide materials accessible for chemical reactions activated by solar radiation, the oxide Ti:1Ru, obtained by the sol–gel method, calcined at 800 °C and subjected to PAHT, stands out for its significant band gap reduction, potentially improving its photoexcitation properties, with a possible activation of 1.56 eV and visible absorption around 795 nm. Alternatively, aiming for an optimum point of economy in the preparation, both modified oxides Nb:1Ru and Ti:1Ru calcined at half the temperature and, after PAHT, offered a band gap around 2 eV and visible absorption around 620 nm.

## Conclusions

In this work, the sol–gel methodology combined with PAHT proved to be an effective and versatile approach for the development and enhancement of titanium and niobium oxides as well as their ruthenium-modified counterparts. The investigations successfully demonstrated that PAHT is a powerful technique for inducing significant changes in the structural, morphological, and optical properties of these materials. A key finding is the promotion of more stable crystalline phases at considerably lower calcination temperatures than those reported for conventional methods. XRD analyses confirmed the formation of stable phases after PAHT—such as rutile for TiO_2_ and monoclinic (H-Nb_2_O_5_) for Nb_2_O_5_—suggesting that the treatment induces pore formation, enhances surface area, and allows for morphology control. In agreement with these structural findings, SEM images revealed well-defined crystalline facets and particle shapes, and EDS analyses and EDX mapping confirmed the homogeneity of the elements expected in the material. PAHT promoted more distinct granules in ruthenium-doped oxides synthesized at higher temperatures. Raman spectroscopy analysis further corroborated these findings, highlighting the evolution of crystal structures in response to the specific synthesis conditions, modifications, and applications of PAHT. The optical and electronic properties were also profoundly influenced. Discussions regarding the inhibition or increase of vacancies were consistent with the sample colors measured by UV–vis DRS. The Ti:1Ru 800 aP oxide was identified as the darkest and most oxidative, which exhibited a remarkable reduction in its band gap energy and a significant increase in visible light absorption. Similarly, the Nb:1Ru 800 oxide showed a striking color change after PAHT, corresponding to its shift to a more thermodynamically stable crystalline phase. These results indicate that the combination of ruthenium doping and PAHT is a highly efficient strategy for stabilizing crystalline structures while simultaneously modulating the optical response of the oxides. The enhanced visible light absorption, particularly in the Ti:1Ru sample, presents promising characteristics for applications in chemical reactions activated by solar radiation such as photocatalysis. Overall, this study contributes significantly to the advancement of knowledge in materials science, establishing PAHT as a valuable tool for the fine-tuning of semiconductor oxides and opening new perspectives for the rational design of materials with specific enhanced functionalities for diverse technological applications.

## Experiments

The oxides were synthesized by an original sol–gel methodology using the raw materials: niobium pentachloride (NbCl_5_, CBMM); isopropyl alcohol (ISOP, Êxodo científica, Sumaré, São Paulo, Brazil); Tween 20 (Synth); titanium­(IV) isopropoxide (Sigma-Aldrich, São Paulo, Brazil); hexaammineruthenium­(III) chloride (Sigma-Aldrich, São Paulo, Brazil); ammonium hydroxide (NH_4_OH, Êxodo científica, Sumaré, São Paulo, Brazil); and Ultrapure water (UPW). The mass of NbCl5 and the volume of titanium isopropoxide were varied to obtain catalysts in the molar proportions Ti/Nb of 100:0 and 0:100 and finalized in a mixture of molar ratio NH_4_OH/M = 2:1 (where M is the sum of Ti^4+^ and Nb^5+^ ions). The precursor catalysts were also modified by adding hexamine ruthenium­(III) chloride in a 1% m/m proportion. After 72 h of standing at room temperature, the precipitate was filtered, washed with UPW, and dried at 100 °C. The heating ramp used for two levels of calcination temperature (400 °C: 800 °C) was 1 °C min^–1^, with the temperature kept constant for 30 min at every 100 °C and 5 h when reaching the desired temperature level. Therefore, eight solid solution oxides were synthesized, as shown in Table 2. The oxides synthesized had their structure characterized by XRD analysis using a diffractometer (X’Pert PRO MPD, PANanalytical) equipped with Cu Kα radiation and Bragg–Brentano geometry. The voltage and current employed were 30 kV and 10 mA, respectively. The crystalline phases could be identified by comparing the obtained diffractograms with the inorganic crystal structure database (ICSD). Besides, the oxides synthesized had their surface evaluated by SEM (Vega 3 LMU, Tescan) and EDS (AZTec Energy X-Act, Oxford). An EDX system was coupled to the SEM. Furthermore, Raman spectra (Xplora Plus, Horiba) were obtained using a 532 nm diode laser excitation coupled to a CCD camera. The measurements were obtained using a 50× objective, with 30 s of integration time at three different regions of the sample. There were no significant differences between the different regions, and the average spectra for each surface were calculated. Finally, the estimated band gap energy and color of the developed oxides were evaluated by ultraviolet–visible (UV–vis) diffuse reflectance spectrometer (Cary 50, Varian) equipped with a Czerny-Turner monochromator, xenon (Xe) lamp, and dual silicon (Si) diode detectors in the region 360 to 830 nm, simulating natural daylight (CIE D65 illuminant) at a 10° angle. Table 1 shows the color of the oxides. The six basic oxides in powder form ranged from very light, subtle gray to light gray, and of the six Ru-modified oxides, from light brown to very dark brown. PAHT made the oxides lighter, with emphasis on the oxide Nb:1Ru 800.

The synthesized and characterized oxides then underwent pressure-assisted heat treatment (denoted PAHT) under rigorously controlled conditions to optimize their properties. Each oxide was packaged in a sterilized glass container and then placed in a hermetically sealed chamber specially designed for this method. The initial air pressure within the chamber and the chamber temperature were simultaneously increased. PAHT was performed in an environment with a constant pressure of 2.1 MPa and a temperature of 110 °C and maintained for a period of 36 h. The air pressure was evenly distributed within the chamber, which allowed for better pressure action on each particle of the oxide subjected to PAHT.

## Supplementary Material



## Notes

Rietveld refinement for bases and modified oxides; SEM images and EDX elemental maps of O, Ti, or Nb, and Ru for the base oxides of TiO_2_, Nb_2_O_5_, and ruthenium-modified oxides without and after PAHT; and EDS spectrum for bases and modified oxides after PAHT (PDF)
