# Supplementary material for: Effect of Pressure-Assisted Heat Treatment on the Crystalline Phase of Titanium Dioxide, Niobium Pentoxide, and Ruthenium-Modified Oxides
Source: ACS Omega. 2026 Jan 23;11(5):7680–90. doi: 10.1021/acsomega.5c08955 (PMC12903154; doi:10.1021/acsomega.5c08955)
Supplement: Supplementary file 1 [file ao5c08955_si_001.pdf]

Support Information

Effect of Pressure-Assisted Heat Treatment on the Crystalline Phase of Titanium Dioxide, Niobium Pentoxide, and Ruthenium-Modified Oxides.

Pietra B. Pires[1], *Maria E. K. Fuziki*[1], *Giane G. Lenzi*[1,2], *Simone do Rocio F Sabino*[2], *Andressa Novatski*[3], *Sergio M. Tebcherani*[4], *Daniele Toniolo Dias*[*,2,5]

[1]Universidade Tecnológica Federal do Paraná – Campus Ponta Grossa, Departamento Acadêmico de Engenharia Química, Ponta Grossa, PR, 84017-220, Brazil.

[2]Universidade Tecnológica Federal do Paraná – Campus Ponta Grossa, Centro de Caracterização Multiusuário em Pesquisa e Desenvolvimento de Materiais, Universidade Tecnológica Federal do Paraná, Ponta Grossa, PR, 84017-220, Brazil.

[3]Universidade Estadual de Ponta Grossa, Departamento de Física, Ponta Grossa, PR, 84030-900, Brazil.

[4]Universidade Tecnológica Federal do Paraná – Campus Ponta Grossa, Programa de Pós-Graduação em Engenharia de Produção, Ponta Grossa, PR, 84017-220, Brazil.

[5]Universidade Tecnológica Federal do Paraná – Campus Ponta Grossa, Departamento Acadêmico de Física, Ponta Grossa, PR, 84017-220, Brazil.

Corresponding Author

*Email: danieletdias@utfpr.edu.br

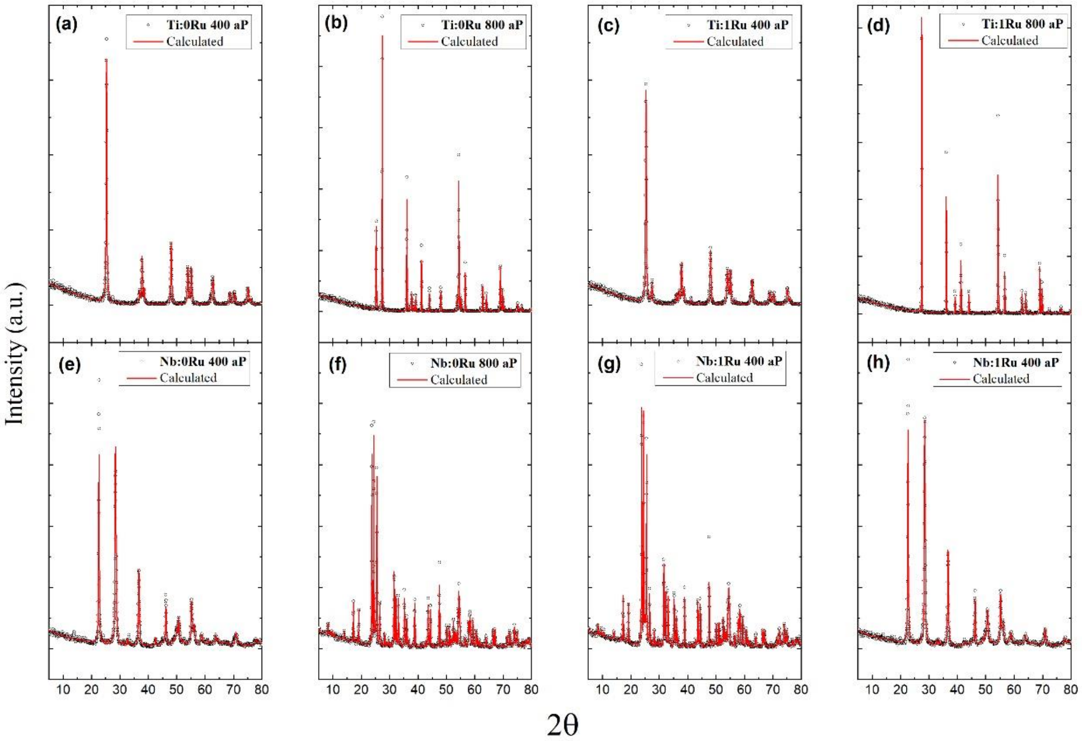

Figure S1: Rietveld refinement for the base oxides of TiO_2, Nb_2O_5, and ruthenium-modified oxides, typically, after pressure-assisted heat treatment. (a), and (e) Calcined base samples, i.e., Ti:0Ru, and Nb:0Ru at 400ºC of calcination temperature. (b), and (f) Base samples at 800ºC of calcination temperature. (c), and (g) Calcined modified samples, i.e., Ti:1Ru, and Nb:1Ru 400ºC of calcination temperature. (d), and (h) Modified samples at 800ºC of calcination temperature.

Table S1. Complementary parameters obtained after Rietveld Refinement.

Sample Code	alpha (°)	beta (°)	gamma (°)	χ^2
**Ti:0Ru 400 wP**	90	90	90	1.849
**Ti:0Ru 400 aP**	90	90	90	1.672
**Ti:0Ru 800 wP**	90	90	90	2.044
	90	90	90	
**Ti:0Ru 800 aP**	90	90	90	1.621
	90	90	90	
**Ti:1Ru 400 wP**	90	90	90	1.525
	90	90	90	
**Ti:1Ru 400 aP**	90	90	90	1.412
	90	90	90	
**Ti:1Ru 800 wP**	90	90	90	2.384
**Ti:1Ru 800 aP**	90	90	90	2.172
**Nb:0Ru 400 wP**	90	90	90	4.095
**Nb:0Ru 400 aP**	90	90	90	2.94
**Nb:0Ru 800 wP**	90	119.811	90	1.567
**Nb:0Ru 800 aP**	90	119.827	90	1.79
**Nb:1Ru 400 wP**	90	90	90	3.464
**Nb:1Ru 400 aP**	90	90	90	2.692
**Nb:1Ru 800 wP**	90	90	90	3.768
**Nb:1Ru 800 aP**	90	119.823	90	2.118

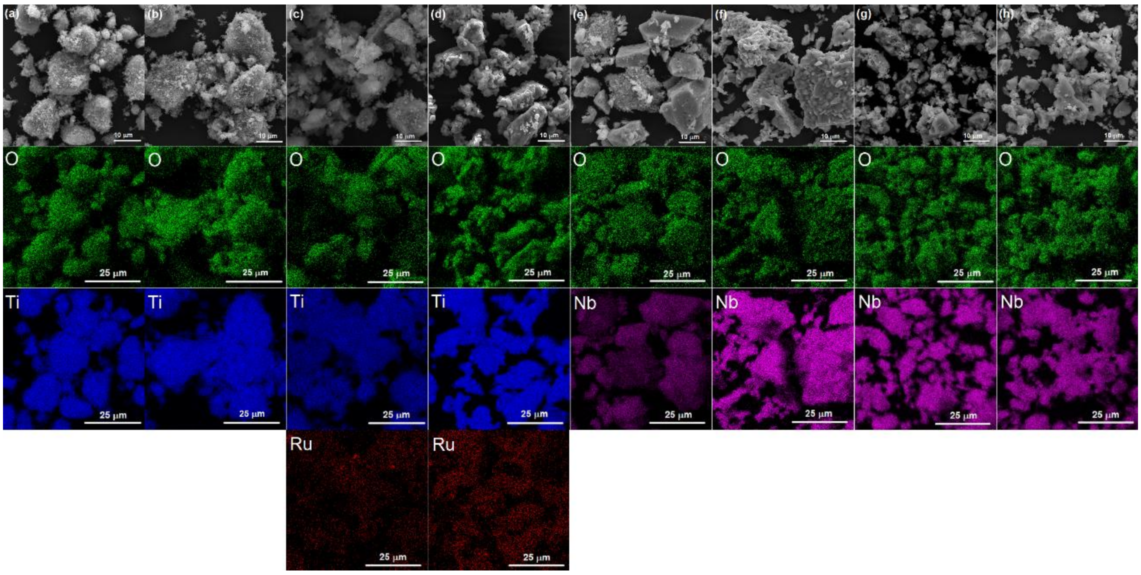

Figure S2. SEM images for the base oxides of TiO_2, Nb_2O_5, and ruthenium-modified oxides without pressure-assisted heat treatment, showing EDX elemental maps of: O (green); Ti (blue) or Nb (magenta); and Ru (red). (a), and (e) Calcined base samples, i.e., Ti:0Ru, and Nb:0Ru at 400ºC of calcination temperature. (b), and (f) Base samples at 800ºC of calcination temperature. (c), and (g) Calcined modified samples, i.e., Ti:1Ru, and Nb:1Ru 400ºC of calcination temperature. (d), and (h) Modified samples at 800ºC of calcination temperature. The element ruthenium is difficult to detect in a niobium pentoxide matrix by EDX primarily due to the higher background signal and potential peak overlaps associated with the heavier Nb matrix.

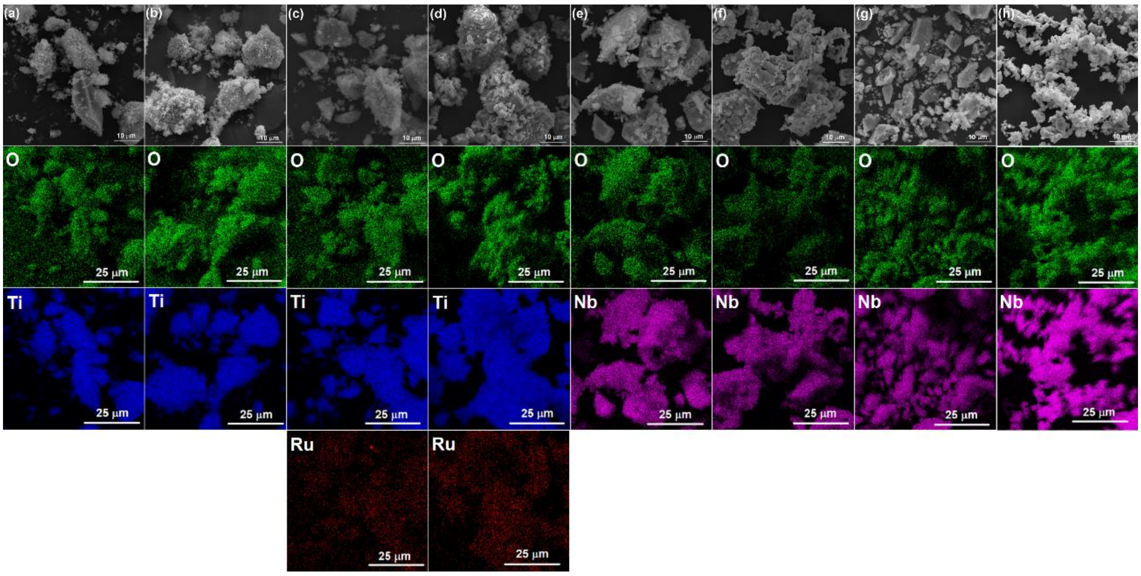

Figure S3. SEM images for the base oxides of TiO_2, Nb_2O_5, and ruthenium-modified oxides after pressure-assisted heat treatment, showing EDX elemental maps of: O (green); Ti (blue) or Nb (magenta); and Ru (red). (a), and (e) Calcined base samples, i.e., Ti:0Ru, and Nb:0Ru at 400ºC of calcination temperature. (b), and (f) Base samples at 800ºC of calcination temperature. (c), and (g) Calcined modified samples, i.e., Ti:1Ru, and Nb:1Ru 400ºC of calcination temperature. (d), and (h) Modified samples at 800ºC of calcination temperature. The element ruthenium is difficult to detect in a niobium pentoxide matrix by EDX primarily due to the higher background signal and potential peak overlaps associated with the heavier Nb matrix.

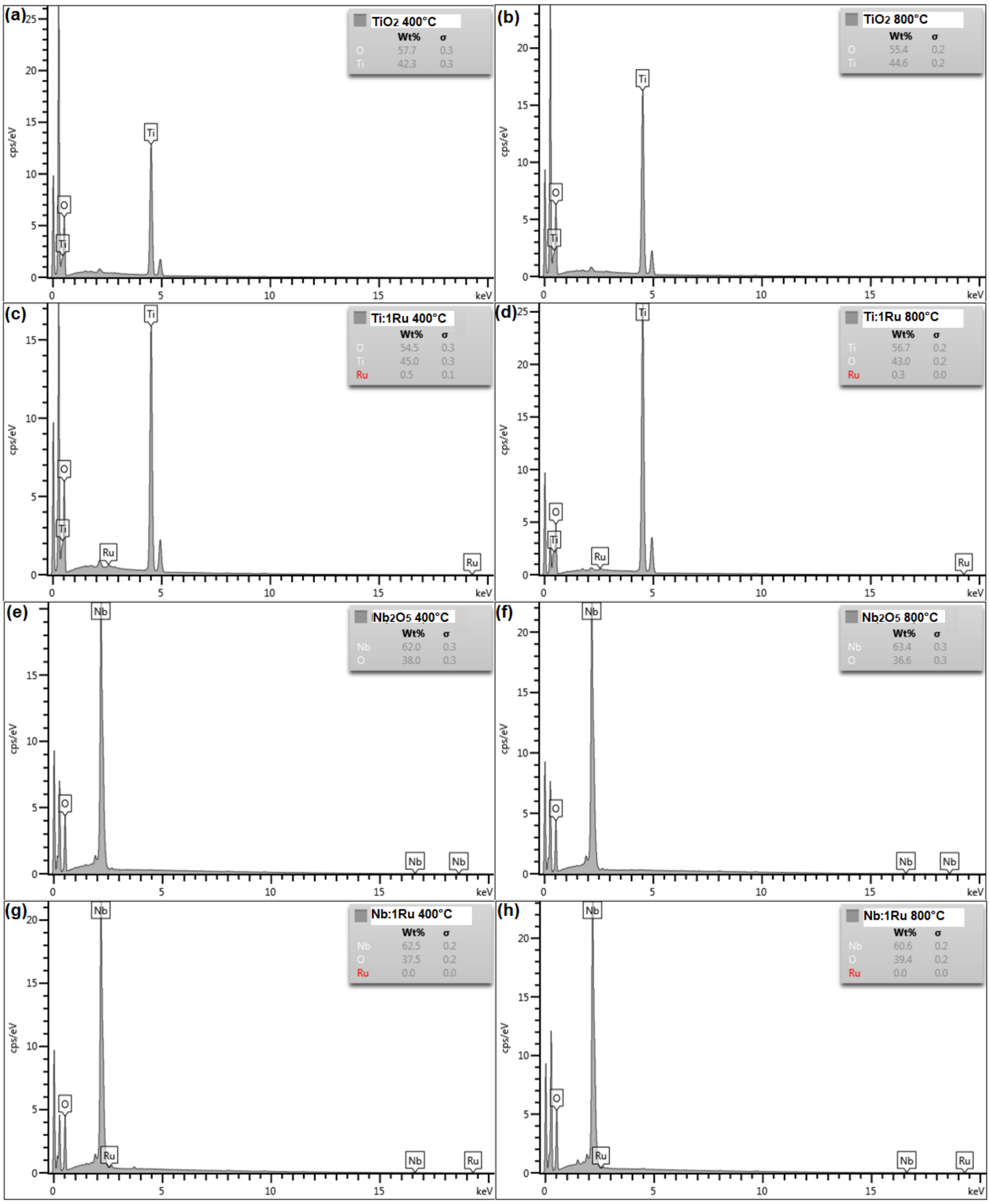

Figure S4. EDS spectrum of calcined base and modified samples after PAHT, i.e., Ti:0Ru, Ti:1Ru, Nb:0Ru, Nb:1Ru at: (a), (c), (e) and (g) 400°C of calcination temperature, and (b), (d), (f) and (h) 800°C of calcination temperature.
